# Supplementary material for: Fear of an unprecedented, invisible enemy: Difficulties experienced in establishing criteria for the release of COVID-19 patients from isolation in a Japanese University Hospital
Source: PLoS One. 2022 Apr 14;17(4):e0266853. doi: 10.1371/journal.pone.0266853 (PMC9009610; doi:10.1371/journal.pone.0266853)
Supplement: Attachment — Submitted filename: Response to Reviewers.docx [file pone.0266853.s002.docx]

10th/February/2022

*Dr. Kensaku Kasuga*

*Academic Editor*

*PLOS ONE*

Ref: PONE-D-22-01171-R1

Fear of an Unprecedented, Invisible Enemy: Difficulties Experienced in Establishing Criteria for the Release of COVID-19 Patients from Isolation in a Japanese University Hospital

We hereby resubmit our above-named manuscript for reconsideration for publication in *PLOS ONE*. We have carefully considered all of the enclosed comments and addressed them as thoroughly as possible. Point-by-point responses to the reviewers’ comments are given below. The corrected sentences are noted with track changes in the revised version.

We hope you will now find our revised manuscript finally acceptable for publication in *PLOS ONE*.

Sincerely yours,

Hideharu Hagiya, M.D., Ph.D.

Department of General Medicine, Okayama University Graduate School of Medicine, Dentistry and Pharmaceutical Sciences, 2-5-1 Shikata-cho, Kita-ku, Okayama 700-8558, Japan

Tel: +81-86-235-7342	Fax: +81-86-235-7345

E-mail: [hagiya@okayama-u.ac.jp](about:blank)

**Comment from Reviewer #1**

Hideharu Hagiya et.al. performed a Google Form based questionnaire survey regarding the requirement, level of understanding, and readiness for developing a criterion of the release of COVID19 patients from in-hospital isolation to general wards. Their major finding was that more than half of health care workers not managing COVID19 patients are not in trouble in the absence of such a criterion while the others actually managing COVID19 are in trouble. To be honest, this obvious finding is not adding any scientific novelty, however this research is still worth publishing on the point highlighting the conflict in medical workers on the COVID19 management. The following point should be addressed for acceptance.

**Response**

We greatly appreciate your effort to review our study. We have provided point-by-point comments below.

Introduction section

1. Please clarify what kinds of preventive measure are required for diseases of “a category 2 infectious disease according to the Act on the Prevention of Infectious Diseases and Medical Care for Patients with Infectious Diseases” in Japan

**Response**

Thank you for your question. In Japan, for a patient with these diseases, a prefectural governor can order hospitalization for the purpose of infection prevention and control. I have added this point into the introduction. (Page 4)

2. Briefly summarize “the discharge protocol for COVID19 patents” required by the Japanese government.

**Response**

We have additionally explained the isolation rule for COVID-19 patients in Japan as follows; “*At that time, the Japanese government set the isolation period for 10 days after the disease onset, if 3 days have passed since the series of symptoms were alleviated.”* (Page 4)

3. Also show “the criteria for in-hospital release from isolation” you made.

**Response**

Thank you for your suggestion again. We have revised and given the new sentences that explain the “the criteria for in-hospital release from isolation” in Page 5.

Result section

4. How many HCWs received request for this questionnaire survey? If the responder rate is relatively small (e.g. less than 50%), description about selection bias should be placed in the limitation paragraph.

**Response**

It is difficult to count up the exact number of people receiving our proposal on the survey. The number was estimated around 2,000, which was added in the methods section. Then this point was added as one of limitation points. (Page 6 and 11)

5. I am also afraid the numbers of HCWs treating COVID19 patients (22.7%) seems a little high. The authors should clarify whether “HCWs treating COVID19 patients” means “HCWs currently treating COVID19” or “including HCWs who used to treat COVID19, but now works in general wards”.

**Response**

We appreciate your comment. We meant “HCWs who were treating COVID-19 patients at the time of the survey“. The corresponding part was revised as such. (Page 7)

6. It is possible that HCWs treating COVID19 patients tend to replay more actively than those in general wards. Comparing responder rates between the groups would be informative.

**Response**

It is now impossible to figure out the responding rates between the two category responders. However, at the time of survey, we set the Google survey not to accept repeated answers from the same identification. We consider this could prevent the reply you are worrying about. We addressed this point in the method section. (Page 6)

Discussion section

7. “The tendency not to express individual opinions in public may be a peculiarity among Japanese people”

It would be better to add a reference to support above description.

**Response**

We have given the recent article regarding this sentence. (Page 10)

**Comment from Reviewer #2**

This study investigated about difficulties encountered in attempting to establish a common understanding of the management of emerging infections. they concluded The dissemination of their experiences and multifaceted discussions with HCWs would be of great value as a countermeasure against the emergent pandemic.

this is well conducted study.

**Response**

We greatly appreciate your effort to review our study. Thank you for your comment.
